# Real-World Cataract Surgery Complications and Secondary Interventions Incidence Rates: An Analysis of US Medicare Claims Database

**DOI:** 10.1155/2022/8653476

**Published:** 2022-04-06

**Authors:** Daniel Terveen, John Berdahl, Mukesh Dhariwal, Qian Meng

**Affiliations:** ^1^Vance Thompson Vision Clinic, Omaha, USA; ^2^Alcon Vision, LLC, Fort Worth, USA; ^3^Trinity Partners, LLC, Waltham, USA

## Abstract

**Purpose:**

To characterize cataract patients and postoperative outcomes in the Medicare fee-for-service (FFS) population.

**Design:**

A retrospective observational cohort study.

**Methods:**

Medicare fee-for-service (A&B) databases were queried from October 2015 to December 2017. Patients with procedural claims using CPT codes (66982 or 66984) and with 1–12 months of postcataract follow-up data were included in the analysis.

**Results:**

133,896 records of 82,246 CMS FFS claims were included in the analysis. The average patient age was 73.8, and 58.2% were females. The cataract surgery setting was ASC (71.3%) followed by HOPD (27.6%). The median time between first and second surgery was 15 days. The most common comorbidities included diabetes (28.6%), glaucoma (22.1%), and macular degeneration (21.7%). Posterior capsule rupture occurred in 0.2% of cases. The <6 months cumulative incidence of most common secondary surgical interventions was 4.7%, 0.2%, and 0.2% for Nd:YAG capsulotomy, IOL exchange, and IOL repositioning, respectively. *Discussion*. Real-world complication rates of cataract surgery may help reduce postcataract complications and procedure burden. *Synopsis for Table of Contents*. This study focused on a sample of the US Medicare beneficiary cataract population and describes its demographic characteristics and reports the cumulative incidence of common postcataract surgery complications and secondary surgical interventions (SSI).

## 1. Introduction

Globally, cataracts account for 33% of treatable blindness in adults [1]. Cataracts are the most common cause of vision impairment for adults in the United States [2]. Prevalence may be as high as 60% in those 75 years or older [3]. This disease burden is significant given the shifting demographics of the United States with a growing proportion of adults over the age of 65. In 2010, the National Eye Institute estimated the overall prevalence of cataracts to be 17.11% and predicted that 38 million and 50 million people will have cataracts in 2030 and 2050, respectively [4].

Despite such projections, there is a total absence of studies examining patients undergoing cataract surgery since the full implementation of International Classification of Disease, Tenth Revision (ICD-10) codes. As such, the present study focused on recent Medicare claims of these patients to identify the most prevalent postoperative complications within one-year postsurgery and to characterize patients undergoing cataract surgery. In addition, it reports the cumulative incidence of common postcataract surgery complications and secondary surgical interventions (SSI).

Given the gaps in the literature, this paper will help identify postcataract complications and SSIs and examine changing patterns in patient characteristics due to demographic changes. These analyses will help provide insight into the potential burden of cataracts on patients and their caretakers, surgeons, and the healthcare system. This study will also help identify implications for new technological advancements in cataract surgery as the patient population ages to help surgeons in optimizing postoperative outcomes.

## 2. Methods

We conducted a retrospective observational cohort study of Medicare beneficiaries undergoing cataract procedures. This study did not require IRB approval as Medicare parts A and B are publicly available datasets, and patient data are deidentified.

### 2.1. Data Source

Data came from the Medicare Limited Data Set (LDS) fee-for-service parts A and B databases claim between October 1, 2015, and December 31, 2017, provided by the Centers for Medicare and Medicaid Services (CMS) [5]. The dataset includes 100% of the fee-for-service institutional claims (hospitals, nursing facilities, etc.). Noninstitutional fee-for-service claims (ambulatory surgical centers, physician offices, physicians, physician's assistants, nurse practitioners, and other standalone providers) were provided by CMS for a random sample of 5% of patients.

### 2.2. Patients

Patients who had procedural claims with at least one cataract surgery current procedural terminology code (either CPT code 66982 or 66984) during the study period (10/1/2015–12/31/2017) were included in the study. The index data for all patients were the date of their cataract surgery. A 6-month precataract period was used to examine baseline characteristics, including comorbidities, and to ensure that patients did not have a prior cataract surgery claim for the same eye.

Inclusion criteria were as follows:

  ≥ 1 procedural claims with at least one cataract surgery current procedural terminology code during the study period
  Continuously enrolled for 6 months before cataract surgery
  Continuously enrolled for at least 1 month up to 12 months following cataract surgery

Exclusion criteria were as follows:

  Patients with Medicare advantage part C
  Patients dual-eligible for Medicare and Medicaid
  Patients with traumatic cataract

### 2.3. Measures and Analysis

Measures included patient characteristics, comorbidities, and postoperative complications. Patient characteristics included age, ethnicity (reported by Medicare beneficiary), sex, census region, surgical setting (inpatient, outpatient, ambulatory surgical center, physicians' claims), and the average time between first and second eye cataract surgery for those with bilateral surgery. We also analyzed the distribution of implanted intraocular lenses (IOLs) (standard monofocal IOL, toric IOL, and presbyopia-correcting IOL).

Comorbidities measured included 18 total comorbid conditions of interest, and the Charlson Comorbidity Index (CCI) score was recorded for all patients. The CCI score is a weighted index based on several conditions that are assigned an integer weighted value from one to six, with the weight of six representing the most severe morbidity. In this study, the CCI scores were measured over the 6 months before surgery [6]. All diagnoses were identified using the ICD-10 codes [7].

Lastly, postoperative complications were recorded at ≤1 day, ≤7 days, ≤30 days, ≤90 days, ≤180 days, and ≤1 year postsurgery. Intraoperative complications included cumulative posterior capsule rupture rate. In addition, the incidence of ocular postoperative complications was analyzed. Secondary surgical interventions analyzed included cumulative incidence of Nd:YAG capsulotomy, IOL exchange, and IOL repositioning at each defined follow-up time-point.

Descriptive statistics of patient demographics, comorbidities, intraoperative, and postoperative complications and secondary surgical interventions were calculated.

## 3. Results

### 3.1. Patient Characteristics

A total of 133,896 eligible cataract surgery records were identified from 82,246 patients (Table 1). The mean patient age was 73.8 years (95% CI 73.7–73.8). The majority (90%) of the patients was between 65 and 84 years of age, and 58.2% of the patients were female. Patient ethnicity was overwhelmingly white (89.8%), and geographically, most patients were from either the South (41.3%) or Midwest (23.3%). Patients from the west and northeast were split evenly in the population (17.9% and 17.2%, respectively). Most procedures (71.3%) took place in ambulatory surgical centers and hospital outpatient settings (27.6%), whereas none of the procedures took place in hospital inpatient settings. The median and the average days between the first and second surgery for patients with bilateral surgery were 15 and 33 days, respectively. The distribution of IOL type by the total procedure count was as follows: standard monofocal IOL (*n* = 128,776), toric IOL (*n* = 3,179), and presbyopia-correcting IOL (*n* = 1,957).

### 3.2. Comorbidities

Common comorbidities among patients were diabetes (28.6%), glaucoma (22.1%), and macular degeneration (14.2%) (Table 2). All other comorbidities were present in less than 10% of patients. Among all patients in the study, the mean CCI score was 1.63. The most common CCI scores for patients were 0 (38.1%) and 1–2 (38.1%) indicating having none or mild comorbidities. Approximately 9% of patients had a CCI score ≥5, indicating they suffered from severe comorbidities.

### 3.3. Postoperative Complications (Table 3 and Table 4)

Posterior capsular rupture occurred in 0.2% of cases (*n* = 314), and endophthalmitis occurred in 0.1% of cases.

The most common postoperative complication one year after surgery was posterior capsule opacification (PCO) where 21.3% of cases experienced PCO ≤1-year postsurgery based on their ICD-10 diagnosis (H26.491, H26.492, H26.493, H26.40). Stratified by IOL, the postoperative cumulative incidence of PCO ≤1 year was most common in presbyopia-correcting IOL (36.3%), followed by the toric IOL (23.9%) and monofocal IOL (21.1%) subgroups. PCO grading was not coded in the patient claims; therefore, it was not possible to ascertain if the PCO was clinically significant or nonsignificant.

Across the entire study cohort, the six-month and one-year cumulative incidence rates of Nd:YAG laser capsulotomy were 4.7% and 8.7%, respectively. Stratified by IOL subtypes, the six-month YAG capsulotomy cumulative incidence rate was 4.7% in monofocal IOLs, 6.5% in toric IOLs, and 13.7% in presbyopia-correcting IOL subgroups. Similarly, the one-year postoperative YAG capsulotomy cumulative incidence rate stratified by IOL subtypes was 8.5% in monofocal IOLs, 11.3% in toric IOLs, and 22.0% in presbyopia-correcting IOLs.

IOL repositioning increased slightly over time, with 0.1% of cases ≤1 day after surgery and 0.2% of cases ≤6 months after surgery. Stratified by IOL types, the IOL repositioning cumulative incidence was highest in the toric IOL (0.9%) subgroup, followed by the presbyopia-correcting IOL (0.8%) and standard monofocal IOLs (0.2%) subgroups. Analysis at a follow-up of ≤1 year was not possible due to an insufficient number of cases in the toric and presbyopia-correcting IOL subgroups. The two most common reasons for IOL repositioning, based on an analysis of ICD codes, were displacement of IOL (T85.22XA) (35.9%) and mechanical complications of IOL (T85.29XA) (10.1%).

Cumulative incidence of IOL exchange postsurgery was 0.2% ≤ 6 months after surgery. Stratified by IOL types, IOL exchange cumulative incidence was highest in the presbyopia-correcting IOL (0.8%) subgroup, followed by the toric IOL (0.5%) and standard monofocal IOL (0.2%) subgroups. As with the IOL repositioning analysis, an analysis of complications for ≤1 year was not possible due to an insufficient number of cases in the presbyopia-correcting IOL subgroup. The two most common ICD codes among IOL exchange claims included mechanical complication of IOL (27.4%) and displacement of IOL (13.5%).

## 4. Discussion

This study used Medicare parts A and B fee-for-service (FFS) claims data to identify cataract patients seeking surgery and their baseline characteristics, comorbidities, postoperative complications, and secondary surgical intervention rates.

Consistent with previous studies, we found that patients with certain demographic characteristics were at a higher incidence of cataract surgery. The average patient of the Medicare population in this analysis was female, white, from a southern state, and 74 years old, which is consistent with other studies that have identified similar demographic characteristics of cataract patients [8, 9]. A study by Schein et al. (2012) presented descriptive demographic characteristics of Medicare fee-for-service cataract patients and found that the rate of surgery was higher among females and those who were white [9]. Additionally, a previous study by Chen et al. described Medicare patients undergoing cataract surgery. The average patient age at the time of cataract surgery was reported to be 76.1 years, which was slightly older than patients in this study (73.8 years) [10]. These types of demographic characteristics are important when examining shifts in population in the United States for the future, and the potential burden that these patients who develop cataracts may incur on the Medicare system.

The most common comorbidities identified in the analyzed population (diabetes, glaucoma and macular degeneration) are consistent with previous studies examining the Medicare population undergoing cataract surgery. In a study by Stein et al. (2011), the researchers found that approximately, a third of patients (29.1%) had diabetes, which is similar to our finding of 28.1%. Another study found the prevalence of diabetes to be 22.1% among Medicare beneficiaries undergoing cataract surgery [11]. Tseng et al. (2012) found the prevalence of glaucoma to be 19.1% which is close to the reported rate in this study (22.1%) [12]. Additionally, most patients in our study had CCI index scores that were below two, which is similar to the distribution noted by Tseng et al. (2012) [12].

In our study, we found that the most common postoperative complication was PCO with a 1-year incidence rate of 21.3%. Retinal edema had an incidence rate of 4.5% while the rest of the complications had incidence rates that were <1% one year following surgery (Table 3). Other studies have looked at the incidence of postoperative complications and secondary surgical interventions in the Medicare population and found similar results [11, 13]. A study by French and Margo (2012) reported a 90-day incidence of secondary procedures per 1,000 surgeries in right and left eyes in the Medicare population similar to our results in Table 3 [14]. The researchers reported 90-day rates of CPT-coded secondary procedures per 1,000 surgeries (in 2007). In this period, YAG incidence rate was 0.90% (right) and 0.82% (left), IOL exchange was 0.05% (right) and 0.06% (left), and repositioning of IOL was 0.18% (right) and 0.26% (left). The reported complication of posterior capsule rupture rate in our study was 0.2%. We used the concurrent claim of an anterior vitrectomy alongside cataract surgery as the basis for estimating capsule rupture rate which may have led to underreporting of data. Incidence of capsule rupture/tear that was managed without the need for anterior vitrectomy procedure was likely not reported in the procedural claims. However, our finding for posterior capsule rupture rate is not vastly different from the rate (0.37%) reported by the CMS Quality Reporting Center [15].

### 4.1. Strengths and Limitations

Our study has several strengths. First, CMS fee-for-service data allowed us to analyze a very large sample size, and as shown previously, it can be generalized to the US cataract population [13]. To our knowledge, no other studies have conducted this type of research upon implementation of ICD-10 codes in the Medicare claims, which enables researchers to determine the laterality of eye undergoing the procedure and experiencing postoperative complications and/or secondary surgical interventions adding to greater precision in estimating the incidence of postoperative events.

This study has some limitations too. First, we chose data from 2015 to 2017 as the study period despite longer data availability in Medicare. We chose a more recent time frame to ensure consistent implementation of ICD-10 codes, consistency in determining laterally of the eye (left/right) undergoing cataract surgery as well as postoperative complications, and to study recent trends in complications and patient characteristics that can inform current practice and policy.

Second, as other researchers have noted, there may be limitations inherent to Medicare claims data and the potential for coding errors [11, 13]. Patients with the Medicare Advantage plan, or those dual-enrolled, did not appear in the fee-for-service dataset. With these patients excluded, this may have produced a biased assessment of the patient's comorbidities, healthcare resource utilization, and costs. Medicare fee-for-service database does not capture claims information on individuals enrolled in private insurance, other government-sponsored insurance, or uninsured individuals. Like others suggested, coding errors, like those for comorbidities, may not capture disease severity and mortality risk factors [11]. For example, patients with diabetes could be at higher risk of developing macular edema postcataract surgery; however, we could not analyze this association in our study due to the possibility of coding errors and omissions. We suggest that future research linking deidentified patient electronic health records and claims datasets should be conducted to assess such causal associations.

Finally, we included only the Medicare population as data for patients with cataract surgery coverage by a private/employer insurance were not available to us for analysis. However, more than 70% of all cataract procedures are performed in the Medicare population, so we believe our analysis can be considered largely representative of the US cataract population [9].

The presence of a cataract can deteriorate a patient's quality of life as visual impairment reduces one's independence in performing daily tasks, worsens one's health, and negatively affects overall well-being [16]. The loss of visual acuity is associated with reduced physical functioning in the elderly population including limitations on mobility and daily activity [17]. Cataracts can also increase the risk of falls, car accidents, and contribute to nursing home placement [18–20]. These outcomes place a significant burden on patients and their caretakers and contribute to the indirect cost of this disease. The high economic burden for the healthcare system, patients, and healthcare providers remains another negative effect of cataracts. Cataract surgery results in improvements in the quality of life for patients, including cognition and mental health, and ultimately is estimated to result in savings to the health system [21, 22]. A study by the Eye Research Institute estimated that the 13-year net financial return on investment from a 1-year cataract surgery cohort of Medicare patients was $36.4 billion, and a 13-year return on investment was over $100 billion “from a 1-year cohort after-cataract surgery”, which includes an increase of productivity in the U.S. of over $25 billion [22]. Over the 13-year model, first-eye cataract surgery conferred a quality-adjusted life-year (QALY) of 1.612, and the bilateral cataract surgery yielded 2.812 QALYs over the same period of time [22]. Given the national average CMS payment for an uncomplicated cataract surgery ranges from $1,587 (ASC) to $2,627 (HOPD) [23] and significant patient benefits postsurgery, cataract surgery is a highly cost-effective procedure with substantial improvement in patient's quality of life [24].

Given these indirect and direct consequences of cataracts and the significant patient benefits of cataract surgery, we hope this study will provide relevant insights into cataract patient demographic characteristics, comorbidities, and their postsurgical outcome to surgeons to optimize surgical outcomes and reduce the overall burden.

## 5. Conclusion

Medicare fee-for-service patients undergoing cataract surgery follow a particular demographic pattern with certain comorbidities and postoperative complications. Understanding these patterns provides insight into cataract patient characteristics. Our results may also help surgeons understand recent trends in the incidence of postoperative complications and inform patients of potential risks. Continuous advancements in cataract surgery technologies could further enable surgeons in reducing postoperative complications. This will hopefully further reduce the postoperative clinical burden and result in ultimate cost-savings for Medicare and patients.[25]

## Figures and Tables

**Table 1:** Demographic characteristics of patients with cataract diagnosis in the Medicare fee-for-service population.

	Cataract diagnosis	
	Number	Percent
Total number of procedures	133,896	
IOL implants		
Claims by cataract surgery (66982)		
Claims by cataract surgery (66984)		
Procedures by setting of care		
Outpatient	36,942	27.60%
Ambulatory surgical center	95,426	71.30%
Other carrier claims	1,528	1.10%
Total number of patients	82,246	
Age, mean (95% CI)	73.76 (73.71, 73.80)	
Age group		
<65	3,010	3.6%
65–74	44,560	54.1%
75–84	29,054	35.4%
≥85 years	5,622	6.80%
Sex		
Female	47,858	58.20%
Male	34,388	41.80%
Ethnicity		
Asian	803	1.00%
Black	4,541	5.50%
Hispanic	439	0.50%
Native American	348	0.40%
White	73,892	89.80%
Others	1,285	1.60%
Unknown	938	1.10%
Census region		
Midwest	19,141	23.30%
Northeast	14,150	17.20%
South	33,981	41.30%
West	14,731	17.90%
Others	196	0.20%
Unknown	47	0.10%

**Table 2:** Comorbidities of Medicare fee-for-service patients undergoing cataract surgery.

Comorbidity^1^	Number	Percent
Cataract	82,235	100.0
Diabetes	23,553	28.6
Glaucoma	18,139	22.1
Macular degeneration	11,720	14.2
Age-related macular degeneration	6,162	7.5
Diabetic retinopathy	2,402	2.9
Miosis	2,030	2.5
Floppy iris syndrome	1,667	2.0
Macular hole	1,136	1.4
Vitreous haemorrhage	526	0.6
Proliferative diabetic retinopathy	505	0.6
Pupillary abnormality	408	0.5
Fracture	370	0.4
Diabetes with ophthalmic manifestations	337	0.4
Corneal edema	312	0.4
Posterior synechiae of iris	262	0.3
Other anomalies of pupillary function	199	0.2
Other disorders of iris and ciliary body	142	0.2
Ocular trauma	20	0.0

^1^Comorbidities were measured during the 6-month preoperative period. Percentages do not add to 100% given that patients may have multiple comorbidities.

**Table 3:** Postoperative ocular complications in Medicare fee-for-service patients who have undergone cataract surgery.

Complication group	Days postcataract surgery					
	≤1 day	≤7 days	≤30 days	≤90 days	≤180 days	≤1 year
Posterior capsular rupture	0.20%	N/A	N/A	N/A	N/A	N/A
Posterior capsular opacification†	0.40%	0.50%	1.20%	3.50%	10.90%	21.30%
Retinal edema	0.20%	0.30%	1.10%	2.60%	3.60%	4.50%
Retinal tear	0.00%	0.10%	0.20%	0.40%	0.70%	1.00%
Retinal detachment	0.10%	0.10%	0.20%	0.50%	0.70%	1.00%
Retained cataract lens fragments	0.10%	0.20%	0.40%	0.50%	0.60%	0.60%
Intraocular lens implant malfunction	0.00%	0.10%	0.20%	0.30%	0.40%	0.40%
Posterior synechiae of iris	0.20%	0.20%	0.20%	0.20%	0.20%	0.30%
Vitreous prolapse	0.10%	0.10%	0.10%	0.20%	0.20%	0.30%
Revision or repair of operative wound of anterior segment	0.00%	0.00%	0.10%	0.10%	0.10%	0.20%
IOL prosthesis secondary implant	0.10%	0.10%	0.10%	0.10%	0.10%	0.20%
Endophthalmitis	N/A	0.00%	0.10%	0.10%	0.10%	0.10%
Hypotony	N/A	0.00%	0.00%	0.00%	0.10%	0.10%
Repair of iris or ciliary body	0.00%	0.00%	0.00%	0.00%	0.00%	0.10%

^†^Includes after-cataract obscuring vision and after-cataract not obscuring vision; excludes Soemmering's ring. N/A indicates insufficient records available to conduct the analysis

**Table 4:** Incidence of secondary surgical interventions postcataract surgery in the Medicare fee-for-service population IOLs.

Follow-up time-point ≤6 months				
	# IOL implants	Cumulative incidence of YAG capsulotomy (≤6 months)	Cumulative incidence of IOL repositioning (≤6 months)	Cumulative incidence of lens exchange (≤6 months)
Overall study cohort (all IOL subtypes)	99,375	4.70%	0.19%	0.24%
Monofocal IOL subgroup	95,579	4.52%	0.16%	0.23%
Astigmatism correcting IOL subgroup	2,345	6.48%	0.85%	0.51%
Presbyopia correcting IOL subgroup	1,451	13.71%	0.76%	0.83%

Follow-up time-point ≤12 months				
	# IOL implants	Cumulative incidence of YAG capsulotomy (≤12 months)	Cumulative incidence of IOL repositioning (≤12 months)	Cumulative incidence of lens exchange (≤12 months)
Overall study cohort (all IOL subtypes)	59,519	8.70%	NA	NA
Monofocal IOL subgroup	57,347	8.46%	0.19%	0.24%
Astigmatism correcting IOL subgroup	1,367	11.27%	NA	0.80%
Presbyopia correcting IOL subgroup	805	22.00%	NA	NA

NA: not available due to insufficient event records. N.B. : data could not be analyzed for the overall cohort if it was missing in one or more subgroups.

## Data Availability

The Medicare Limited Data Set used to support the findings of this study is available at https://www.cms.gov/Research-Statistics-Data-and-Systems/Files-for-Order/LimitedDataSets.

## Abbreviations

ASC: Ambulatory surgical centers
CCI: Charlson comorbidity index
CMS: Center for Medicare Services
FFS: Fee for service
HOPD: Hospital outpatient department
IOL: Intraocular lens
LDS: Limited data set
Nd YAG laser: Neodymium-yttrium-aluminum-garnet laser
PCO: Posterior capsular opacification
SSI: Secondary surgical interventions.

## Additional Points

Value statement: *What Was Known.* Untreated cataracts are a source of economic and patient burden in the Medicare population in the United States. There is a need to understand the characteristics of patients at risk and those undergoing surgery to prevent future burden and reduce postoperative complications. *What This Paper Adds*. Medicare patients undergoing cataract surgery follow a specific pattern with regard to their demographics and comorbidities, which makes these surgery candidates predictable and identifiable. Postoperative complications also follow a clear pattern, with the most common complications being PCO and associated secondary surgical intervention of Nd:YAG capsulotomy. In the future, the authors can minimize the risk of complications through technological evolution. This will reduce the postoperative clinical burden and result in cost savings for Medicare.
